# Role of miRNA-mRNA Interaction in Neural Stem Cell Differentiation of Induced Pluripotent Stem Cells

**DOI:** 10.3390/ijms21196980

**Published:** 2020-09-23

**Authors:** Satish Kumar, Joanne E. Curran, Erica DeLeon, Ana C. Leandro, Tom E. Howard, Donna M. Lehman, Sarah Williams-Blangero, David C. Glahn, John Blangero

**Affiliations:** 1 Department of Human Genetics and South Texas Diabetes and Obesity Institute, University of Texas Rio Grande Valley School of Medicine, McAllen, TX 78504, USA; erica.deleon02@utrgv.edu (E.D.); sarah.williams-blangero@utrgv.edu (S.W.-B.); 2 Department of Human Genetics and South Texas Diabetes and Obesity Institute, University of Texas Rio Grande Valley School of Medicine, Brownsville, TX 78520, USA; joanne.curran@utrgv.edu (J.E.C.); ana.leandro@utrgv.edu (A.C.L.); tom.howard@utrgv.edu (T.E.H.); john.blangero@utrgv.edu (J.B.); 3 Department of Medicine, University of Texas Health Science Center at San Antonio, San Antonio, TX 78229, USA; lehman@uthscsa.edu; 4 Department of Psychiatry, Boston Children’s Hospital and Harvard Medical School, Boston, MA 02115, USA; david.glahn@yale.edu; 5 Olin Neuropsychiatric Research Center, Institute of Living, Hartford, CT 06102, USA

**Keywords:** human, induced pluripotent stem cell, neural stem cell, miRNA, mRNA, gene expression regulation

## Abstract

miRNA regulates the expression of protein coding genes and plays a regulatory role in human development and disease. The human iPSCs and their differentiated progenies provide a unique opportunity to identify these miRNA-mediated regulatory mechanisms. To identify miRNA–mRNA regulatory interactions in human nervous system development, well characterized NSCs were differentiated from six validated iPSC lines and analyzed for differentially expressed (DE) miRNome and transcriptome by RNA sequencing. Following the criteria, moderated *t* statistics, FDR-corrected *p*-value ≤ 0.05 and fold change—absolute (FC-abs) ≥2.0, 51 miRNAs and 4033 mRNAs were found to be significantly DE between iPSCs and NSCs. The miRNA target prediction analysis identified 513 interactions between 30 miRNA families (mapped to 51 DE miRNAs) and 456 DE mRNAs that were paradoxically oppositely expressed. These 513 interactions were highly enriched in nervous system development functions (154 mRNAs; FDR-adjusted *p*-value range: 8.06 × 10^−15^–1.44 × 10^−4^). Furthermore, we have shown that the upregulated miR-10a-5p, miR-30c-5p, miR23-3p, miR130a-3p and miR-17-5p miRNA families were predicted to down-regulate several genes associated with the differentiation of neurons, neurite outgrowth and synapse formation, suggesting their role in promoting the self-renewal of undifferentiated NSCs. This study also provides a comprehensive characterization of iPSC-generated NSCs as dorsal neuroepithelium, important for their potential use in in vitro modeling of human brain development and disease.

## 1. Introduction

The cell fate determination during embryonic development and adult tissue homeostasis is orchestrated by global changes in the cellular transcriptome and proteome—a complex but tightly regulated process involving diverse regulatory mechanisms. MicroRNAs (miRNAs) are ~22-nucleotide-long, noncoding RNAs that regulate the posttranscriptional expression of protein coding genes [1,2]. The regulatory process typically involves complement base-pairing of the 5-prime region of the mature miRNA known as the seed sequence to the 3-prime untranslated region (3’ UTR) of complementary/partially complementary messenger RNAs (mRNAs). The binding of the miRNA with cognate mRNAs typically results in either the destabilization or suppressed translation of the mRNA targets [3,4]. This complementary nature of the miRNA–mRNA interaction is being exploited to computationally predict and identify miRNAs regulatory targets [5,6,7,8].

Like transcription factors, single miRNAs can regulate the expression of numerous genes and a single protein coding gene can be a target of multiple miRNAs. The miRNAs spatial and temporal expression is tightly regulated by cellular developmental and physiological state, indicating a key role that miRNAs play in regulating cell fate and differentiation [3,9]. Complex miRNA regulatory events are woven into the known transcription factor and signaling networks that control cell fate and differentiation, modulating their activity through positive and negative feedback loops to reinforce cellular decisions. However, miRNA expression is highly cell- and tissue-specific [3,10,11].

During embryonic development, pluripotent embryonic stem cells give rise to fate-restricted multipotent stems cells, which, in response to specific stimuli, can commit to a given cellular fate while retaining a broad self-renewal potential. Neural stem cells (NSCs) are encountered in both the embryonic and adult brain and are capable of differentiating into all cell types of the central nervous system. During development, different intra- and extracellular signals stimulate NSCs to become neural progenitors, which eventually irreversibly exit the cell cycle to begin the first stage of neurogenesis. However, before this event occurs, the self-renewal and proliferative capacities of neural stem cells must be tightly regulated, and miRNAs may play a key role in maintaining the neural stem cell state as well as in the developmental processes of the nervous system [11].

During the last one and half decades, human embryonic stem cells (ESCs) and human-induced pluripotent stem cells (iPSCs) in particular, have been exploited as a valuable cell system which carries the intrinsic capability of indefinite self-renewal and the potential to model the tissue-specific physiology through the use of differentiation protocols to generate specific target cell/tissue types [12]. The tissue-specific differentiation of human ESCs and iPSCs has also been a valuable model to study the early steps of human development. The acquisition of tissue-specific cell state by ESCs and iPSCs through in vitro differentiation protocols follows a sequential activation of gene expression program and epigenetic changes that closely mimic the developmental events occurring during early embryogenesis [13,14,15,16].

The human iPSC differentiated NSCs have the transcriptional profile of dorsal neuroepithelium (discussed in the results section), an early stage of brain development that gives rise to the majority of the central nervous system, and therefore represents a relevant cell type to model early neurodevelopmental and associated disorders [17]. To better understand the role of miRNA and mRNA interactions in regulating iPSC to NSC differentiation as well as the role of such interactions in the maintenance of neural stem cell state, in this manuscript, we have analyzed genome-wide miRNA and mRNA sequencing data generated from iPSCs and their differentiated NSCs of six different individuals.

## 2. Results

### 2.1. Differentiation and Lineage-Specific Functional Characteristics of Generated NSCs

**Figure 1:**
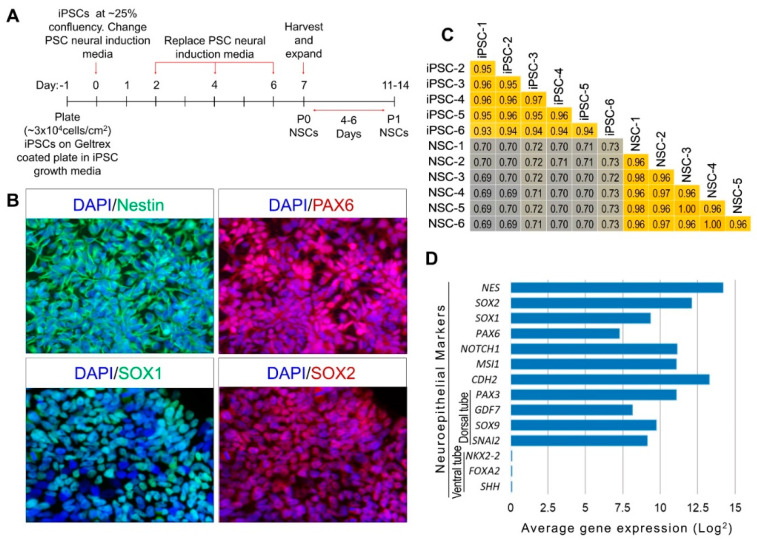
Characterization of human-iPSC generated NSCs by immunocytochemistry (ICC) and gene expression analysis. (**A**) Schematic diagram of iPSC to NSC differentiation. (**B**) ICC analysis of generated NSCs showing expression of NSC markers Nestin, PAX6, SOX1 and SOX2. (**C**) Correlation coefficient (*r*^2^) plot based on all expressed mRNAs between six iPSCs and their differentiated NSCs. (**D**) Gene expression plot showing dorsal neuroepithelial transcriptomic profile of generated NSCs.

The NSC differentiation of validated iPSC lines, generated from six different individuals, as described in our previous publications, Kumar et al. [18,19], were induced using the Gibco PSC neural induction medium (Thermo Fisher Scientific, Waltham, MA, USA) and method as described in Yan et al. [17], and summarized in the schematic presented in Figure 1A. An extensive characterization of generated NSCs on day 14 (at passage P1) was performed using immunocytochemistry (ICC) analysis of the NSC-specific markers and by genome-wide mRNA sequencing. The iPSC differentiated NSCs expressed the NSC markers Nestin, PAX6, SOX1 and SOX2 across all six samples (Figure 1B). The average correlation coefficient at 95% CI between six NSC samples, calculated based on all expressed mRNAs, was 0.97 ± 0.008, suggesting a uniform differentiation of generated NSC lines (Figure 1C). The generated NSCs showed a high expression of neuroepithelial genes/transcription factors (*NES*, *SOX2*, *SOX1*, *PAX6*, *NOTCH1*, *MSI1* and *CHD2*) and genes/transcription factors of dorsal tube neuroepithelium (*PAX3*, *GDF7*, *SOX9* and *SNAI2*) but lacked the expression of ventral tube neuroepithelial markers (*NKX2-2*, *FOXA2* and *SHH*), suggesting a dorsal tube neuroepithelial transcriptomic and functional profile of the generated NSCs (Figure 1D). Our iPSC differentiated NSCs showed epithelial-like characteristics at a higher confluence of monolayer culture.

**Figure 2:**
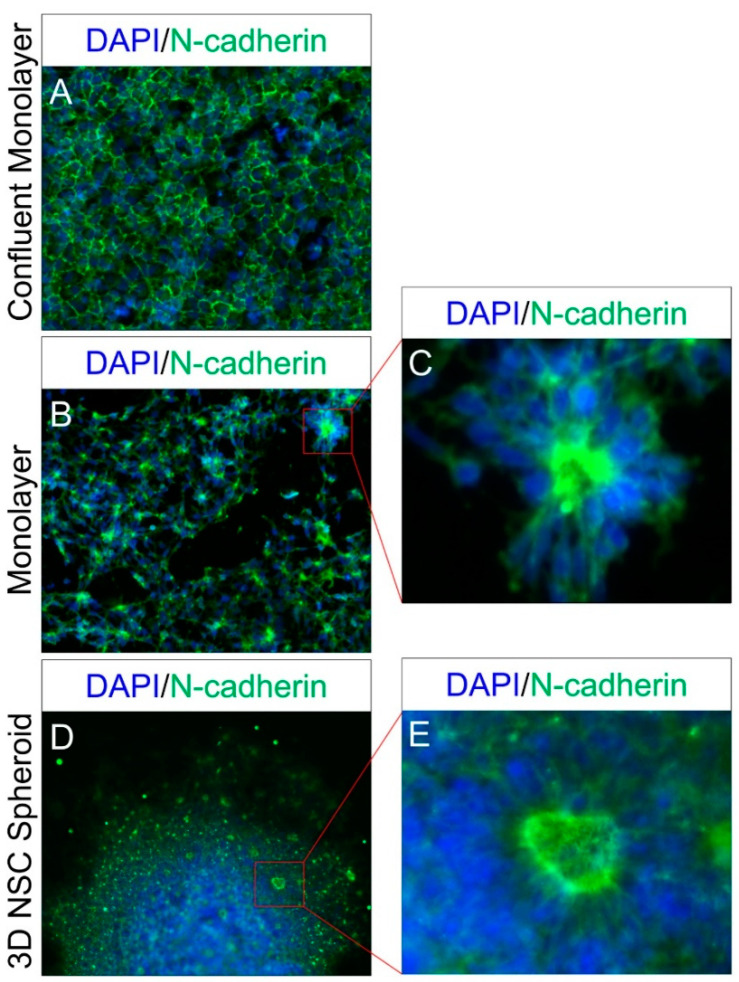
Apical-basal polarity characteristic of iPSC-generated NSCs. (**A**) Epithelial cells like localization of adherens junction’s protein N-cadherin in confluent NSC monolayer culture. (**B**,**C**) Acquisition of radial apical basal property and N-cadherin localization in NSC monolayer culture. (**D**,**E**) Self-organization of differentiated NSCs into neural rosettes like structures in a 3D NSC spheroid culture.

Therefore, we further tested the generated NSCs for the apical basal polarity characteristics of the neuroepithelial cells by the ICC localization of the adherens junction’s protein N-cadherin, both in NSCs cultured in a monolayer at a lower confluence, as well as by generating 3D NSC spheroids. The localization of N-cadherin in both monolayer and 3D NSC spheroid cultures showed that the generated NSCs had the inherent apical-basal characteristics of neuroepithelial cells and were capable of self-organization into neural rosette like structures within the cultured 3D NSC spheroids (Figure 2). 

### 2.2. Differentially Expressed (DE) Genes

The miRNA and mRNA sequence data generated from six iPSC and their differentiated NSC lines were submitted to the gene expression omnibus (GEO) archive (accession numbers GSE74289 and GSE156617). A total of 8.3 and 6.9 million miRNA 40 bp single-end reads and 32.2 and 517.9 million mRNA 100bp paired-end reads were obtained for iPSCs and their differentiated NSCs, respectively.

**Figure 3:**
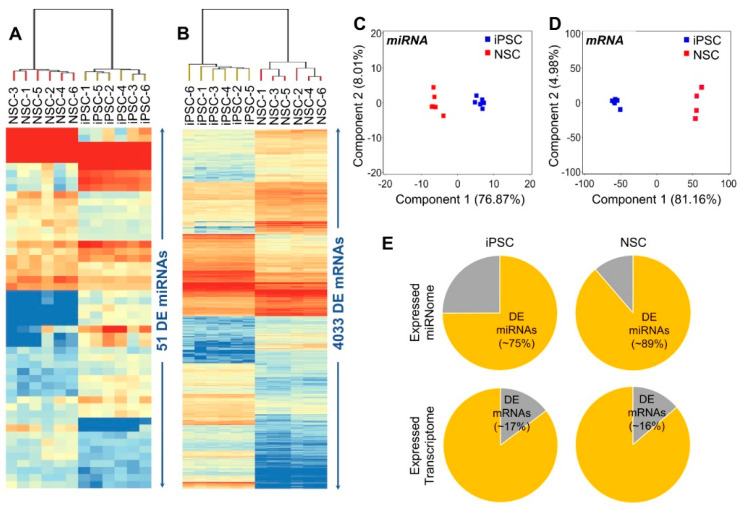
miRNA and mRNA differential expression analysis between iPSCs and their differentiated NSCs. (**A**) Heat map of 51 differentially expressed (DE) miRNAs. (**B**) Heat map of 4033 DE mRNAs. (**C**,**D**) Principal component analysis (PCA) based on DE miRNAs and DE mRNAs between iPSCs and differentiated NSCs. (**E**) Pie graph showing the proportion of DE vs. total expressed miRNome and transcriptome in iPSCs and their differentiated NSCs.

The iPSCs and their differentiated NSCs showed a greater global overlap in their expressed transcriptome (correlation coefficient at 95% CI = 0.71 ± 0.010), likely due to shared characteristics such as self-renewal. However, expression heat maps and principal component analysis of DE miRNAs and mRNAs between iPSCs and differentiated NSCs showed a discrete and uniform resetting of both miRNome and transcriptome during these cellular transitions (Figure 3A–D). About 77% of the variance in DE miRNA’s expression and ~81% of the variance in DE mRNA’s expression can be attributed to the NSC differentiation of iPSCs (Figure 3C–D).

DE miRNAs: As indicated above, 51 miRNAs were found significantly DE between iPSCs and their differentiated NSCs and accounted for nearly 75% of the iPSC’s and 89% of the NSC’s expressed miRNome (Figure 3E). The 25 miRNAs, which were significantly down-regulated in differentiated NSCs included miR-302a/b/c/d and miR-371/372/373, miRNA clusters/families associated with human iPSC/ESC pluripotency and maintenance; miR-200 family (miR-200b/c) involved in E-cadherin and N-cadherin regulation, and its down-regulation promotes N-cadherin expression and thus pluripotency to neural transition [20,21]. The miR-92a and miR-92b were highly abundant in iPSCs and showed significant upregulation in differentiated NSCs. The 26 miRNAs, which were significantly upregulated in differentiated NSCs, included the *HOX* gene cluster associated miR-10a/b; miR-30 family (miR-30a, miR-30b, miR-30c); miR-219a-2; miR-130B, which is associated with embryonic neural stem and progenitor cells proliferation; and miR-197, involved in the regulation of NOTCH signaling. 

DE mRNAs: The 4033 mRNAs that were significantly DE between iPSCs and their differentiated NSCs, accounted for nearly 17% of the iPSC’s and 16% of the NSC’s expressed transcriptome (Figure 3E). A total of 2027 of these DE mRNAs were significantly down-regulated in the differentiated NSCs (Table S1) and were highly enriched in mRNAs that are known to be involved in the stemness, pluripotency and self-renewal of human iPSCs and included core pluripotency transcription factors *POU5F1* and *NANOG*, however, the expression of *SOX2*, which also plays a key role in neural induction, did not change significantly. The 2006 DE mRNA were significantly up-regulated in differentiated NSCs (Table S1) and were highly enriched in neuroepithelial markers (discussed above) and developmental functional categories such as embryonic development (572 mRNAs; FDR adjusted *p*-value range: 1.92 × 10^−53^–3.26 × 10^−9^), organism development (749 mRNAs; FDR adjusted *p*-value range: 1.92 × 10^−53^–4.30 × 10^−9^), nervous system development and function (629 mRNAs; FDR adjusted *p*-value range: 3.23 × 10^−48^–4.15 × 10^−9^), tissue development (655 mRNAs; FDR adjusted *p*-value range: 3.63 × 10^-40^–4.32 × 10^−9^) and organ development (406 mRNAs; FDR adjusted *p*-value range: 8.74 × 10^−40^–2.37 × 10^−9^).

### 2.3. In-Silico miRNA Target Prediction and Their Interaction with iPSC-NSC DE Transcriptome

Having established the DE miRNome and transcriptome between iPSCs and their differentiated NSCs, next we sought to determine mRNA targets of the DE miRNAs that may play a regulatory role in NSC differentiation of iPSCs and in early neural development. The miRNA target predictions were performed using TargetScan-Human, TarBase and miRecords databases implemented in Qiagen Ingenuity Pathway Analysis (IPA) platform release June 2020 (Qiagen, Redwood City, CA, USA). The 51 miRNAs that were DE between iPSCs and their differentiated NSCs mapped to 39 mature miRNA families, and 30 of these miRNA families, following the criteria that interaction sites were either experimentally observed and/or predicted with high confidence, were predicted to target 3811 mRNAs, resulting in 4619 total miRNA-mRNA interactions. A total of 793 of these mRNA targets were DE between iPSCs and their differentiated NSCs and resulted in 999 miRNA–mRNA interactions, 456 of these targets were oppositely expressed and resulted in 513 interactions between oppositely expressed miRNA–mRNA pairs (Supplementary Materials Table S2). As per the IPA knowledge base, about 10% of these interactions were experimentally observed in previous studies. About 40% of these 513 miRNA–mRNA interactions were significantly enriched in organism development (209 mRNAs; FDR adjusted *p*-value range: 4.38 × 10^−15^–1.52 × 10^−4^) and tissue development (204 mRNAs; FDR adjusted *p*-value range: 8.06 × 10^−15^–1.52 × 10^−4^) functional categories. About 30% interactions were significantly enriched in nervous system development functions (154 mRNAs; FDR adjusted *p*-value range: 8.06 × 10^−15^–1.44 × 10^−4^). The significantly activated (activation z-score ≥ 1.5) nervous system development functions listed in Table 1 suggest that miRNAs may play a significant regulatory role in the nervous development and differentiation of iPSCs into NSCs.

**Figure 4:**
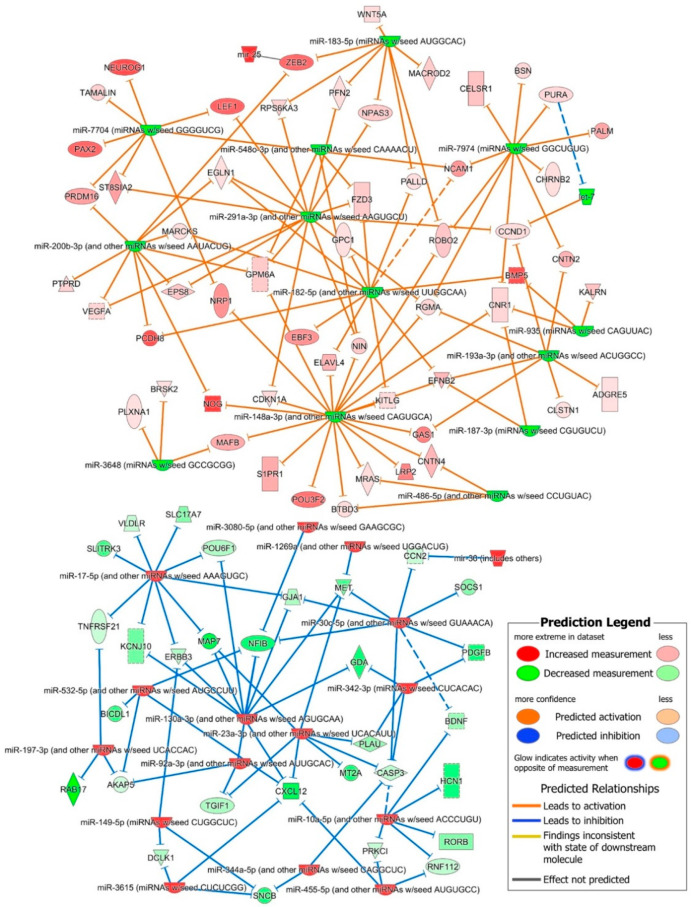
miRNA–mRNA interaction network showing interactions between 85 DE mRNA enriched in significantly upregulated human nervous system development functions and miRNAs found DE between iPSCs and differentiated NSCs.

The 85 mRNAs that were common between these significantly activated nervous system development functions and their predicted interactions with DE miRNAs, shown in Figure 4, have identified several regulatory networks which may play a key role in early neural development. Overall, the upregulated miR-10a-5p, miR-30c-5p, miR-23-3p, miR-130a-3p and miR-17-5p miRNA families were predicted to down-regulate genes that were involved in the specification and differentiation of neurons, neurite outgrowth, synapse formation and neuronal maturation and migration, as well as cellular death, suggesting that these upregulated miRNAs play a role in promoting the self-renewal of undifferentiated NSCs (Figure 4). The downregulated miRNA families miR-291a-3p, miR-200b-5p, miR-182-5p, miR-183-5p, miR-148a-3p, miR-193a-3p, miR-7704 and miR-7974 were predicted to regulated several gene clusters involved in early neural induction of human ESCs and iPSCs, cytoskeleton reorganization and axonal dynamics (Figure 4). Several other miRNA and mRNA interactions inconsistent with the paradoxically opposite expression of miRNA and their targets were also predicted, suggesting that DE miRNA may also play a role in finetuning the expression of such targets in the maintenance of self-renewing NSCs.

### 2.4. Role of DE miRNAs in the Regulation of Key iPSC and NSC Canonical Pathways

Further, we explored the miRNA–mRNA interactions in key canonical pathways that were enriched within the 513 identified interactions and were relevant to iPSC to NSC differentiation and maintenance of self-renewing NSCs.

**Figure 5:**
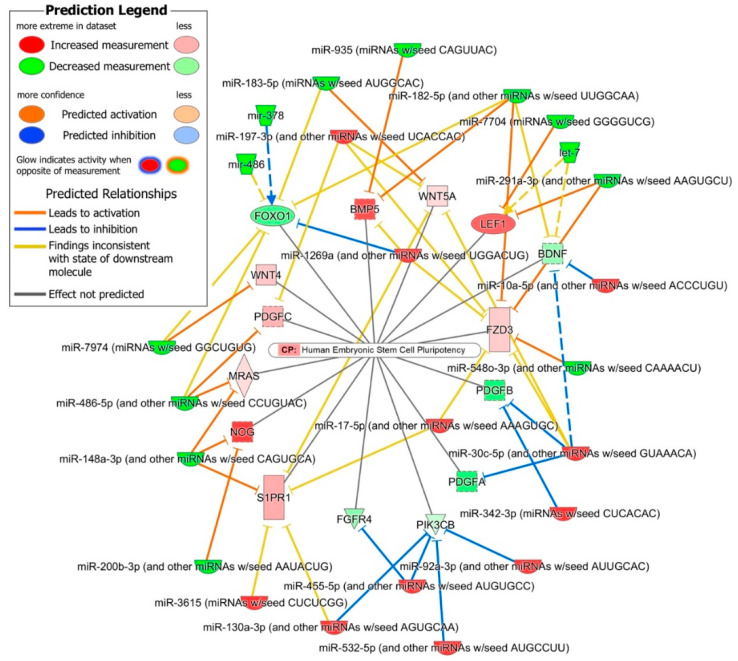
miRNA–mRNA interactions enriched in human ESC pluripotency canonical pathways.

Human ESC pluripotency: The human ESC pluripotency canonical pathway was significantly enriched (*p*-value = 7.59 × 10^−8^) in the 513 miRNA–mRNA interactions predicted between iPSC’s and NSC’s DE miRNome and transcriptome. A total of 17 interactions between oppositely expressed 12 miRNA families and 15 DE mRNAs enriched in human ESC pluripotency canonical pathways were identified. Some interactions between unidirectionally expressed miRNA-mRNA pairs were also observed (Figure 5). Six genes/mRNAs (*FOXO1, FGFR4, PDGFA, PDGFB, PIK3CB* and *BDNF*) associated with iPSC pluripotency and self-renewal that were down-regulated in differentiated NSCs, were the predicted targets of eight upregulated miRNA families (miR-10a-5p, miR-30c-3p, miR-342-3p, miR92a-3p, miR-532-5p, miR-455-5p, miR-1269a and miR-130a-3p). The upregulated Wnt/β-catenin signaling genes/mRNAs *WNT4, WNT5A, FZD3 and LEF1*, and BMP signaling inhibitor *NOG*, *S1PR1, MRAS, PDGFC and BMP5* were the predicted targets of ten down-regulated miRNA families (miR-291a-3p, miR-548o-3p, miR-200b-3p, miR-148a-3p, miR-486-5p, miR-7974, miR-183-5p, miR-182-5p, miR-935 and miR-7704).

**Figure 6:**
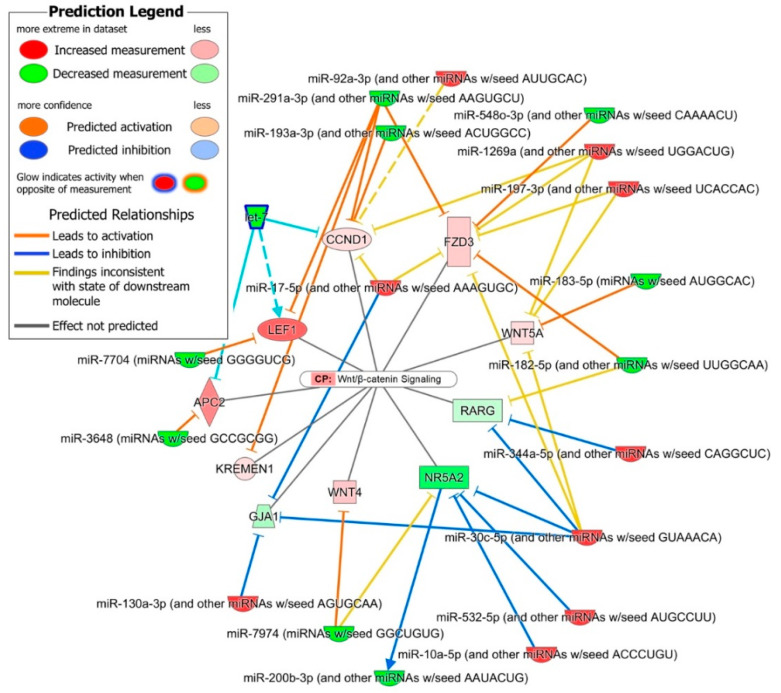
miRNA–mRNA interactions enriched Wnt/β-catenin canonical pathway.

Wnt/β-catenin signaling: As indicated in the human ESC pluripotency pathway, the Wnt/β-catenin signaling genes were significantly enriched (*p*-value = 2.38 × 10^−3^) in the 513 miRNA–mRNA interactions predicted between iPSC’s and NSC’s DE miRNome and transcriptome. The 11 interactions between oppositely expressed 10 miRNA families and 10 DE mRNAs and several between unidirectionally expressed miRNA–mRNA pairs were enriched in the Wnt/β-catenin canonical pathway (Figure 6). The miRNA interactions of four Wnt/β-catenin signaling pathways genes *WNT4, WNT5A, FZD3* and *LEF1*, were discussed in the Human ESC pluripotency pathway above. The *APC2* gene and the two Wnt/β-catenin target genes *CCND1* and *KREMEN1* were predicted targets of four down-regulated miRNA families (miR-291a-3p, miR-193a-3p, let-7 and miR-3648). Nuclear receptor genes *NR5A2* and *RARG* and gene *GJA1* were down-regulated in NSCs and were the predicted targets of five up-regulated miRNA families (miR-10a-5p, miR-532-5p, miR-30c-5p, miR-344a-5p and miR-130a-3p).

## 3. Discussion

We have shown that the NSCs differentiated using the monolayer differentiation protocol and commercially available Gibco PSC neural induction medium (Thermo Fisher Scientific, Waltham, MA, USA), as described in Yan et al. [17], have the characteristics of dorsal neuroepithelium (Figure 1), an early stage of brain development that gives rise to the majority of the central nervous system, and therefore represents a disease-relevant cell type whose production is scalable to a larger sample size required to aid the discovery of molecular and neurodevelopmental changes and the identification of downstream genes or genetic variants influencing the human central nervous system development and disease [12]. Furthermore, the differentiation of these iPSC-generated NSCs into other disease-relevant neural lineage cell types, including cells of ventral neuroepithelium and terminally differentiated neuron types, astrocytes and oligodendrocytes has been robustly demonstrated in our own work, as well as by others [17,18].

The recent advancements in the capability of human ESC/iPSC differentiation strategies to recapitulate major hallmarks of in vivo neural development serve as a valuable resource for modelling the development and disease of the human brain [17,22,23,24,25,26]. Furthermore, better understanding of the regulatory processes involved in differentiation and the regulatory role miRNAs may play in these cellular transitions is critical to improve the in vitro modeling potential of these human ESC/iPSC generated neural cells. However, due the heterogeneity and dynamic nature of the neural progeny, both in in vivo neural development and the iPSC differentiated neural stem/progenitor cells, there is a need to employ differentiation and a stage-specific strategy that allows investigation of these regulatory processes in a uniform NSC population [22,27]. For example, Edri et al. [27] identified five different consecutive stages in the neural rosette-based differentiation of human ESC lines over a period of 220 days of culture. Alternatively, a multipoint time course analysis may be important in identifying the stage-specific changes and regulatory interactions in neural rosette-based differentiation strategies that produce more heterogeneous NSC and progenitor cell populations. Our iPSC-generated NSC expresses *HES5, PAX6* and *SOX1*, along with other progenitor cell markers such as *SOX2* and *NESTIN* (Figure 1D, Table S1) and shares the expression profile marking the establishment of the CNS earliest neuroepithelial cells [28]. The generated NSCs also showed the inherent property to self-organize into radially arranged neural rosettes (Figure 2) and share a very uniform transcriptomic profile across multiple samples (correlation coefficient at 95% CI = 0.97 ± 0.008). Therefore, in this study we have primarily focused on the two well-defined timepoints of neural induction, the embryonic stem cell stage, and the induction/establishment of the CNS earliest neuroepithelial cells, marked by our well-characterized iPSCs and their differentiated NSCs, respectively. 

Our differential expression analysis of the iPSCs and their differentiated NSCs expressed miRNome and transcriptome identified 51 significantly DE miRNAs and 4033 significantly DE mRNAs. As discussed in the results section, the transcriptomic and functional profile of our iPSC-generated NSCs is consistent with the early neuroepithelial cells [17,27]. However the repertoire of 51 DE miRNA between our iPSCs and their differentiated NSCs was smaller than a previous study [29], likely due to some obvious differences in sample size [30,31], differentiation and miRNA typing methodologies and plausibly due to the heterogeneity of the differentiated NSCs in [29], as discussed above. miR-9 and miR-124a, which are known to be involved in differentiation of neural stem and progenitor cells into neurons and astrocytes [3,32,33,34,35], were either not expressed or were expressed below the set expression threshold (NRC < 20) in our NSCs. This further validates the naive neuroepithelial state, also referred to as “primitive” in Yan et al., [17], of the NSCs generated by these methods.

The in silico prediction analysis of the iPSC’s and NSC’s DE miRNome and transcriptome identified 513 interactions between oppositely expressed 30 miRNA families (mapped to 51 DE miRNAs) and 456 DE mRNAs. These 513 predicted miRNA–mRNA interactions were highly enriched in nervous system development functions and the 85 mRNAs common in the significantly activated nervous system development functions (Table 1) and their predicted interactions with DE miRNAs shown in Figure 4 identified several regulatory networks. The upregulated miR-10a-5p miRNA family was the second most abundant in our NSCs after miR-92a-3p and was predicted to regulate the expression of genes, *PRKCI* whose down-regulation promotes NOTCH signaling and NSC self-renewal [36], pro-neural genes *RORB, BDNF* and *RNF112* [37,38,39], and *HCN1* associated with differentiation of sympathetic neurons [40]. miR-10a-5p along with miR-30c-5p and miR342-3p, was also predicted to down-regulate the pro-apoptotic gene *CASP3*. These interactions suggest that the miR-10a-5p family of miRNAs plays a significant role in regulating the self-renewal of undifferentiated NSCs. Similarly, the miRNA families miR-30c-5p, miR23-3p, miR130a-3p and miR-17-5p were predicted to down-regulate genes (Figure 4) involved in the neural specification and differentiation of neurons, neurite outgrowth, synapse formation, and therefore play a role in regulating the differentiation of NSCs [41]. The down-regulated miRNA families miR-291a-3p, miR-548o-3p, miR-7974, miR-183-5p, miR-182-5p, and miR-7704 were predicted to target Wnt/β-catenin associated genes. The up-regulation of the Wnt/β-catenin signaling pathway in NSCs and its down regulation upon neuron differentiation suggests that this pathway may play a role in the self-renewal of undifferentiated NSCs [42]. The BMP signaling inhibitors *NOG* and *ZEB2* were the predicted targets of down-regulated miR-148a-3p, miR-200b-3p and miR-183-5p miRNA families. The E-cadherin suppressor *ZEB* controls the E-cadherin to N-cadherin switch and thus stabilizes N-cadherin-based adherens junctions, which play a critical role in the self-renewal of undifferentiated NSCs [43,44].

## 4. Materials and Methods

The six iPSC lines used in this study were previously reprogrammed from lymphoblastoid cells lines established in-vitro using blood samples of our San Antonio Mexican American Family Study (SAMAFS) participants, who were originally recruited in our San Antonio Family Heart Study (SAFHS), and provided appropriate written consent. The study protocols were approved by the Institutional Review Boards of UT Health San Antonio (HSC20060194H, 16 March 2020) and the University of Texas Rio Grande Valley, Edinburg (IRB-18-0245, 16 March 2020).

### 4.1. NSC Differentiation

A detailed description of iPSC reprogramming and validation methodology used in reprogramming of six iPSC lines and their validation can found in our previous publications [18,19]. The NSC differentiation was induced using commercially available Gibco PSC neural induction medium (Thermo Fisher Scientific, Waltham, MA, USA) and the method as described in Yan et al. [17]. Briefly, iPSCs maintained in feeder-free conditions were split as cell clumps in a Geltrex (Thermo Fisher Scientific, Waltham, MA, USA) coated, six-well plate at a density reaching 15–25% confluence after 24 h and maintained in mTeSR-1 medium (Stem Cell Technologies Inc., Cambridge, MA, USA) supplemented with 10 µM ROCK inhibitor Y27632 (ATCC, Manassas, VA, USA). Approximately 24 h after splitting, culture medium was switched to Gibco PSC Neural Induction Medium (Thermo Fisher Scientific, Waltham, MA, USA). Medium was changed every other day. On day 7, NSCs were dissociated with Stem Pro Accutase (Thermo Fisher Scientific, Waltham, MA, USA) and plated on a Geltrex-coated plate at a density of 1 × 10^5^ cells/cm^2^ in an NSC expansion medium containing 50% Neurobasal medium, 50% Advanced DMEM/F12, and 1X neural induction supplement (all from Thermo Fisher Scientific, Waltham, MA, USA). The NSC expansion medium was supplemented with 5 µM ROCK inhibitor Y27632 (ATCC, Manassas, VA, USA) for first 24 h. NSC expansion medium was changed every other day until NSCs reached confluence. The six generated NSC lines from passage one were used for characterization and total RNA extraction.

### 4.2. ICC-Based Characterization and Apical-Basal Polarity Assay of Generated NSCs

For ICC-based characterization of the iPSC-differentiated NSCs, the cultured NSCs from passage one were fixed using 4% paraformaldehyde (MilliporeSigma, St. Louis, MO, USA) and then immunoassayed using commercially available primary antibodies against NSC specific markers Nestin (Thermo Fisher Scientific, Waltham, MA, USA), PAX6 (Cell Signaling Technology, Inc., Danvers, MA, USA), SOX1 (Thermo Fisher Scientific, Waltham, MA, USA) and SOX2 (Cell Signaling Technology, Inc., Danvers, MA, USA), and standard ICC techniques. 

For apical-basal polarity assays in monolayer culture, the passage one NSCs were plated on a Geltrex-coated plate at a density of 1 × 10^5^ cells/cm^2^ in the NSC expansion medium supplemented with 5 µM ROCK inhibitor Y27632 (ATCC, Manassas, VA, USA) for the first 24 h and then cultured into NSC expansion medium without ROCK inhibitor for the next 24 h. At 48 h, NSC monolayer cultures were fixed with 4% paraformaldehyde (MilliporeSigma, St. Louis, MO, USA) and then immunoassayed using commercially available primary antibodies against adherens junction protein N-cadherin (Thermo Fisher Scientific, Waltham, MA, USA). 

For apical-basal polarity assays in 3D NSC spheroids, 3D NSC spheroids were generated from passage one NSCs (4000 NSCs/spheroid) in an AggreWell 800 plate (Stem Cell Technologies Inc., Cambridge, MA, USA) and maintained in NSC expansion medium for 24 h. After 24 h, the generated 3D spheroids were harvested and plated on laminin-coated plates in an NSC expansion medium. After 120 h, the cultures were fixed with 4% paraformaldehyde (Sigma Aldrich) and then immunoassayed using anti N-cadherin (Thermo Fisher Scientific, Waltham, MA, USA) primary antibodies. 

In each assay, cells were counterstained with 4′,6-Diamidino-2-phenylindole dihydrochloride (DAPI) nuclear stain (MilliporeSigma, St. Louis, MO, USA) and were imaged on a Carl Zeiss epifluorescence equipped inverted microscope (Carl Zeiss Microscopy, LLC, White Plains, NY, USA).

### 4.3. RNA Extraction and Sequencing

Total RNA extraction and small RNA and mRNA sequencing of the six validated reprogrammed iPSCs were performed previously and described in our previous publication [19].

Total RNA extraction: The total RNA from snape frozen cell pellets (~3–5 × 10^6^ cells) from six iPSC differentiated passage one NSCs was extracted using commercially available RNeasy Mini Kit (Qiagen, Germantown, MD, USA) and the manufacturer protocol. RNA quality and quantity were assessed using a NanoDrop 2000 Spectrophotometer (Thermo Fisher Scientific, Waltham, MA, USA) and an Agilent 2200 TapeStation system (Agilent, Santa Clara, CA, USA).

Small RNA Sequencing: Small RNA sequencing libraries were prepared from 1 µg total RNA per sample and using an Illumina TruSeq small RNA sample preparation kit (Illumina, Inc., San Diego, CA, USA). The kit uses a modified RNA 3’ adaptor that specifically targets miRNA and other small RNAs that have a 3’ hydroxyl group. After adaptor ligation to each end, reverse transcription was performed to synthesize complementary single stranded cDNA. The cDNA libraries were then PCR-amplified and gel-purified for a fragment size of 145–160 nucleotides. The size-selected purified cDNA libraries from six NSC samples were then sequenced using the Illumina HiSeq 2500 platform (Illumina, Inc., San Diego, CA, USA).

mRNA Sequencing: The 1 µg total RNA per sample, extracted from six, passage one NSC lines and Illumina TruSeq RNA sample preparation kit v2 (Illumina, Inc., San Diego, CA, USA), was used to prepared mRNA sequencing libraries. Briefly, the poly-A tail containing mRNA molecules was enriched from total RNA using oligo dT attached magnetic beads. The mRNA-enriched samples were then fragmented into ~200–600 base pair fragments using divalent cations under elevated temperature. The cleaved RNA fragments were used as a template to synthesize first-strand cDNA using reverse transcriptase and random primers, followed by second-strand cDNA synthesis, using DNA polymerase-I and RNase H. The synthesized cDNA fragments were then end-repaired, and adaptor ligations were performed. The end-repaired, and adaptor-ligated cDNA libraries were then purified and enriched by PCR before deep sequencing on Illumina HiSeq 2500 platform (Illumina, Inc., San Diego, CA, USA). 

Sequence Analysis: Raw fastq sequence files were generated and demultiplexed using Illumina CASAVA v1.8 pipeline (Illumina, Inc., San Diego, CA, USA). After pre-alignment QCs, sequences were aligned to human genome assembly GRCh38 (hg38) and mapped to RefSeq transcripts using StrandNGS software v3.3 (Strand Life Sciences Pvt. Ltd., Bangalore, India). Small RNA reads were mapped to small RNA annotations inferred from miRbase (v20), ensemble (e75), gtrnadb (tRNA) and UCSC know Gene tables (piRNA) and implemented in Strand NGS software. The aligned reads were filtered based on default read quality matrix and log transformation and “DESeq” normalization were applied. The known miRNAs and mRNAs having an NRC ≥20 in six iPSCs and/or their differentiated NSCs were considered expressed and selected for differential gene expression analysis.

### 4.4. Differential Gene Expression and In-Silico miRNA Target Prediction Analyses

miRNA and mRNA DE analysis: To identify DE miRNAs and mRNAs, moderated *t* statistics and expression fold change analyses were performed on miRNA and mRNA data sets. The miRNAs and mRNAs having moderated *t* statistic FDR corrected *p*-value ≤ 0.05 and FC-abs ≥ 2.0, were considered DE.

miRNA target prediction analysis: The miRNA target prediction analysis was performed using TargetScan-Human, TarBase and miRecords databases as implemented in the Qiagen IPA platform (Qiagen, Redwood City, CA, USA). The TargetScan content in IPA uses TargetScan algorithm-predicted mRNA targets of human miRNAs that are binned into high and moderate confidence. TarBase content identifies experimentally demonstrated miRNA–mRNA interactions from TarBase using miRbase identifiers, and miRecords content uses experimentally validated human, rat, and mouse miRNA-mRNA interactions from the published literature. The mRNA targets predicted with high confidence and experimentally observed mRNA targets from TarBase and miRecords that were significantly DE between iPSCs and their differentiated NSCs and showed paradoxical opposite expression from interacting miRNA(s) were considered for functional analysis.

Functional annotation and gene expression analysis: Functional annotations and pathway analysis were performed using identified miRNA–mRNA interactions between oppositely expressed miRNA and mRNAs and IPA platform. Right-tailed Fisher’s exact test *p* values corrected for FDR were used to calculate enrichment significance. The direction of functional change was assessed by activation Z-score, as implemented in IPA. Further details of the methods implemented in IPA can be found in [45].

## 5. Conclusions

In conclusion, we have identified 51 miRNAs and 4033 mRNAs that were significantly DE between iPSCs and their differentiated NSCs. We have also identified 513 miRNA-mRNA interactions predicted with high confidence between oppositely expressed 30 miRNA families (mapped to 51 DE miRNAs) and 456 DE mRNAs. These 513 interactions were highly enriched in nervous system development functions, suggesting that they may play a key role in regulating early brain development and in the differentiation and maintenance of iPSC-generated NSCs. However, further functional studies are necessary to validate these predicted and potentially regulatory miRNA and mRNA interactions. This study also provides a comprehensive characterization of iPSC-generated NSCs as dorsal neuroepithelium, important for their potential use in in vitro modeling of human brain development and disease.

## Figures and Tables

**Table 1:** The nervous system development functions that were significantly enriched and predicted to be upregulated in 513 miRNA–mRNA interactions identified in iPSCs and their differentiated NSCs.

Nervous System Development Functions	Enrichment *p*-Value	Activation z-Score	Enriched Interaction/mRNAs
Development of neurons	8.06 × 10^−15^	2.074	70
Formation of brain	5.94 × 10^−10^	1.841	44
Development of cerebral cortex	1.63 × 10^−6^	1.969	17
Formation of hippocampus	2.43 × 10^−6^	1.941	13
Neurogenesis of hippocampus	2.78 × 10^−5^	2.219	6
Neurogenesis of brain	4.09 × 10^−5^	2.102	8

## Supplementary Materials

Supplementary materials can be found at https://www.mdpi.com/1422-0067/21/19/6980/s1.
